# Supplementary material for: Collaborative intelligence in AI: Evaluating the performance of a council of AIs on the USMLE
Source: PLOS Digit Health. 2025 Oct 9;4(10):e0000787. doi: 10.1371/journal.pdig.0000787 (PMC12510544; doi:10.1371/journal.pdig.0000787)
Supplement: S2 File — (ZIP) [file pdig.0000787.s002.zip › S2 Data Raw data files containing unprocessed question inputs and LLM outputs including transcripts of deliberations/STEP3_Question-43-1_Type-MC-NJ.docx]

**Facilitator** **[Round 1]:** A 70-year-old man is admitted to the hospital through the emergency department because of a 12-hour history of severe nausea and four episodes of vomiting of undigested food. During the past week, he has had worsening nausea, heartburn following meals, and early satiety. Medical history is significant for type 2 diabetes mellitus diagnosed 20 years ago, hyperlipidemia, and hypertension. Medications are intermediate-acting insulin twice daily and insulin lispro before meals. He also takes enalapril and atorvastatin. On admission, the patient appears uncomfortable. Vital signs are temperature 36.2°C (97.1°F), pulse 102/min, respirations 16/min, and blood pressure 105/78 mm Hg. Pulse oximetry on room air shows an oxygen saturation of 98%. Physical examination discloses mild epigastric tenderness to palpation. The remainder of the examination discloses no abnormalities. Results of laboratory studies ordered in the emergency department are shown: Serum Blood ALT 23 U/L AST 26 U/L Alkaline phosphatase 85 U/L Amylase 104 U/L Urea nitrogen 42 mg/dL Creatinine 1.32 mg/dL Hemoglobin A1c 8.2% X-rays of the chest and abdomen disclose no abnormalities. Which of the following will best explain the patient's condition? (A) Accumulation of calcium carbonate and hemoglobin breakdown products in the bile ducts (B) Decreased gastric myoelectrical activity (C) Decreased gastric pH (D) Increased cholecystokinin activity

**AI_E:** RESTATING THE QUESTION: A 70-year-old man with a history of type 2 diabetes, hyperlipidemia, and hypertension presented with severe nausea, vomiting of undigested food, and other digestive issues. His lab results do not indicate any liver or pancreatic dysfunction, nor do they show any kidney issues. However, the patient's symptoms point to a possible gastrointestinal disorder. We are asked to identify the best explanation for the patient's condition from four options: (A) Accumulation of calcium carbonate and hemoglobin breakdown products in the bile ducts, (B) Decreased gastric myoelectrical activity, (C) Decreased gastric pH or (D) Increased cholecystokinin activity. PLANNING: These options suggest different pathophysiological mechanisms. Option A suggests a biliary tract disease which often presents with symptoms like jaundice or pain, not seen here. Option B implies a motility disorder, which can lead to symptoms like nausea and vomiting. Option C implies a change in stomach acidity, affecting digestion, whereas increased cholecystokinin activity (option D) would refer to increased gallbladder contractions and pancreatic secretions that could lead to symptoms like heartburn or early satiety. CLINICAL REASONING: Considering the patient's history of diabetes, a complication called diabetic gastroparesis might be suspected. Diabetic gastroparesis is a condition in which the stomach cannot empty food properly due to vagal nerve damage caused by long-standing hyperglycemia. This leads to decreased gastric myoelectrical activity, resulting in symptoms such as nausea, vomiting, heartburn, and early satiety. The lab values do not point to an obstruction or stark metabolic disorder, and the symptom profile doesn't clearly align with problems relating to changes in stomach acidity or cholecystokinin function. CONCLUDING: Given the patient's history, his symptoms including recurring nausea and vomiting of undigested food, the condition of diabetic gastroparesis appears the most likely explanation. This condition reflects decreased gastric myoelectrical activity, which would align with Option B. RESPONSE: Therefore, the patient's condition can best be explained by (B) Decreased gastric myoelectrical activity, possibly due to diabetic gastroparesis.

**AI_B:** (1) RESTATING THE QUESTION: The case described presents a 70-year-old male patient who has been experiencing ongoing nausea and vomiting of undigested food, heartburn after eating and feeling full quickly for the past week. He has a significant medical history including type 2 diabetes, high cholesterol, and high blood pressure. His current medications include insulin for his diabetes, and enalapril and atorvastatin for hypertension and hyperlipidemia respectively. Upon hospital admission, an examination revealed that he had mild discomfort around the stomach area. His lab results and x-rays did not present any abnormal findings. The question is asking what is the best explanation for this patient's clinical presentation. (2) PLANNING: This patient has symptoms suggestive of a condition related to the digestive tract, specific to the stomach based on the area being tender during the initial physical examination. The options provided include: accumulation of calcium carbonate and hemoglobin breakdown products in the bile ducts, decreased gastric myoelectrical activity, decreased gastric pH and increased cholecystokinin activity; all of which are associated with different conditions within the gastrointestinal tract. (3) CLINICAL REASONING: To arrive at the most likely explanation, it's beneficial to detail the effects and implications of each option. Option (A) suggests the presence of gallstones which typically manifest with right upper quadrant abdominal pain, fever, and jaundice—this doesn't align with the patient's symptoms. Option (C) infers an increased acidity in the stomach, associated with conditions like GERD or peptic ulcer disease but there are no additional signs (such as weight loss or overt gastrointestinal bleeding) to support these diagnoses. Option (D) increased cholecystokinin activity, typically associated with gallbladder contraction and bile release, would tend to present with postprandial discomfort or tenderness in the right hypochondrium, which doesn't fit the given case. Option (B), decreased gastric myoelectrical activity means a slowdown in the motility or movement of the stomach which could potentially present with symptoms of early satiety, post-meal heartburn, and vomiting of undigested food as the stomach fails to adequately empty its contents. (4) CONCLUDING: Given the above reasoning and factoring the clinical presentation of the patient, option B that is, decreased gastric myoelectrical activity is most likely to explain this patient's condition. This clinical picture is consistent with gastroparesis, a condition often seen in longstanding diabetes where autonomic neuropathy affects the vagus nerve, impairing gastric motility. (5) RESPONSE: The patient's symptoms of severe nausea, vomiting of undigested food, heartburn after meals, and early satiety can best be explained by (B) decreased gastric myoelectrical activity, likely indicative of gastroparesis.

**AI_C:** (1) RESTATING THE QUESTION: You're asking about a 70-year-old man who presented with severe nausea, vomiting of undigested food, worsening heartburn, and early satiety, among other symptoms. He has a significant medical history of type 2 diabetes, hyperlipidemia, and hypertension, and is on specific medications for these conditions. His physical exam and laboratory investigations returned mostly normal results. We have to figure out what could best explain his condition based on the options provided. (2) PLANNING: Let's go over some assumptions based on the options. Option (A) suggests a gallstone-related condition, which is often characterized by right upper quadrant pain, which this patient does not report. Option (B) speaks to a disorder in the stomach's muscular contractions, which could indeed cause issues with nausea, vomiting, and satiety. Option (C) suggests that the patient's stomach acid levels are low, which could arise from various conditions. Option (D) involves increased activity of cholecystokinin, a hormone that aids in digestion but could cause problems if it's too high. Based on the patient's symptoms and history, we could hypothesize that Option (B), decreased gastric myoelectrical activity, might be clinically prioritized. (3) CLINICAL REASONING: Starting with the patient's main complaint of severe nausea, vomiting, and early satiety, these are all signs indicative of a condition affecting the digestive process. His worsening symptoms after meals and long-standing diabete history (especially poorly controlled as suggested by the Hemoglobin A1c of 8.2%) offer additional support for a gastroenteropathy. Diabetes can cause autonomic neuropathy, which affects the nerves in the digestive system leading to gastroparesis - a condition characterized by delayed gastric emptying due to decreased gastric myoelectrical activity. This can explain his symptoms of early satiety, nausea, and vomiting of undigested food, as food stays longer in the stomach. (4) CONCLUDING: Keeping in mind the patient's medical history and the manifesting symptoms, it appears that the most likely explanation for his condition is gastroparesis due to diabetic autonomic neuropathy. His worsening condition following meals, the nature of the vomit (undigested food), and the early satiety are all symptomatic of impaired gastric emptying. The lab results do not indicate any noticeable underlying hepatic, renal, or pancreatic dysfunction. Abdominal x-rays not revealing any abnormalities also rules out anatomical issues as a cause. (5) RESPONSE: Therefore, the possible underlying mechanism best explaining the patient's condition is (B) Decreased gastric myoelectrical activity.

**AI_A:** RESTATING THE QUESTION: The provided information refers to a 70-year-old male patient who was admitted to the hospital due to severe nausea and vomiting of undigested food within the last 12 hours. He has a one week history of progressive nausea, post-meal heartburn, and early satiety. He has medical history of type 2 diabetes, hypertension, and hyperlipidemia. He is on intermediate-acting insulin, insulin lispro, enalapril and atorvastatin. While no abnormalities are detected during physical examination other than mild epigastric tenderness, vital signs and laboratory results are provided. Nothing unusual was detected during X-rays of chest and abdomen. We are asked to choose the option that could best explain his condition. PLANNING: The question is centered around major symptoms such as severe nausea, vomiting of undigested food, heartburn, and early satiety. These symptoms are strongly indicative of a condition related to the digestive system. The options suggest possible pathophysiological mechanisms such as accumulation of certain substances in bile ducts, alterations in gastric myoelectrical activity, changes in gastric pH, and increased activity of cholecystokinin - a hormone that stimulates the release of digestive enzymes from the pancreas and bile from the gallbladder. Given the fact that lab results and X-rays do not provide clear signs of a certain organ failure, this makes the identification of the underlying cause a challenge. But let's evaluate each assumption. CLINICAL REASONING: Let's start by eliminating some options. Cholecystokinin increase (option D) could lead to symptoms relating to biliary colic or pancreatitis, but the patient's lab results (especially amylase level) don't support this. Similar reasoning can be applied to option A, the accumulation of substances in the bile ducts would likely show other symptoms related to biliary tract disease and again, there are no abnormal lab results to suggest this. Also, if there was a major bile duct obstruction, we would expect to see elevated alkaline phosphatase and possibly bilirubin, but these are not present. Now looking at the remaining options: decreased gastric pH (option C) and decreased gastric myoelectrical activity (option B). Decreased gastric pH means increased acidity which could lead to GERD symptoms or even peptic ulcer disease, but typically it would not cause vomiting of undigested food. Given the patient's symptoms, the most likely diagnosis would be gastroparesis, a condition characterized by delayed gastric emptying in the absence of mechanical obstruction. This could result from decreased gastric myoelectrical activity. CONCLUDING: Despite not having a definitive test to confirm, the symptoms experienced by the patient, coupled with his well-controlled diabetes, suggest that gastroparesis due to autonomic neuropathy (impaired myoelectrical activity of the stomach - option B) is a significant assumption to explain his current condition. RESPONSE: The most likely explanation for this patient's condition is (B) Decreased gastric myoelectrical activity which leads to the condition known as gastroparesis. This condition could be a result of longstanding diabetes mellitus which has resulted in autonomic neuropathy.

**AI_D:** A 70-year-old man with a significant history of type 2 diabetes mellitus, hyperlipidemia, and hypertension is currently in hospital with a 12-hour history of severe nausea and vomiting of undigested food. Over the past week, he also experienced worsening nausea, heartburn post-meals, and early satiety. His medication regimen includes intermediate-acting insulin, insulin lispro, enalapril, and atorvastatin. Amplifying the picture, physical examination at the time of admission disclosed mild epigastric tenderness. In contrast, the remaining examination and laboratory investigations demonstrated no perfect abnormalities. The context then asks to identify the most likely explanation for the condition of this patient, offering the following four options: (A) the aggregation of calcium carbonate and by-products of hemoglobin metabolism in the bile ducts; (B) a decrease in gastric myoelectrical activity; (C) a decrease in the pH of the gastric environment; and (D) an increase in cholecystokinin activity. Let's discuss these options in light of the patient's presentation and medical history. Option A - accumulation of calcium carbonate and hemoglobin breakdown products in the bile ducts - generally leads to conditions such as gallstones or in severe cases, can lead to cholestasis. However, the patient's liver function tests are within normal limits, and there are no mentions of any biliary symptoms or findings suggestive of biliary tract disease, which contests this theory. Option C - decreased gastric pH - would suggest an acidic environment, often associated with conditions such as peptic ulcer disease or gastritis. While the patient does complaint of post-meal heartburn, this largely stands unsupported by other clinical or investigative hints. Option D - increased cholecystokinin activity - typically results in gallbladder contraction and pancreatic enzyme release; the absence of signs indicative of gallbladder disease or pancreatitis makes this an unlikely explanation as well. Turning our attention towards the patient's symptoms - severe nausea, vomiting of undigested food, worsening postprandial heartburn, and early satiety - paired with the tenderness noted in the epigastric area, we might recognize these as indicative of a compromise in gastric motility, or gastroparesis. Our patient's longstanding diabetes mellitus – manifested by a HbA1c level of 8.2% - creates a conducive physiological environment for diabetic gastroparesis, a complication that emerges due to potential damage to the autonomic nerves supplying the stomach, leading to disturbed gastric myoelectrical activity and subsequently delayed gastric emptying. Such a clinical picture seamlessly aligns with Option B, earmarking it as our leading hypothesis for the patient's condition. In constructing a conclusion, the most likely explanation for this patient's condition would indeed seem to be decreased gastric myoelectrical activity. The constellation of his presenting symptoms, medical history, and the physical examination findings all hint towards a disturbed gastric motility compatible with diabetic gastroparesis – a scenario which lays the foundation for a decrease in gastric myoelectrical activity. Therefore, (B) Decreased gastric myoelectrical activity is the primary cause of this patient's condition, with his type 2 diabetes mellitus acting as a predisposing factor for the development of diabetic gastroparesis. This aligns with his presentation of nausea, vomiting of undigested food, post-meal heartburn, early satiety, and the absence of abnormal findings in other investigative results.

**Facilitator** The consensus among the AI responses is that the most likely explanation for the 70-year-old male patient's condition is decreased gastric myoelectrical activity (Option B). This conclusion is based on the patient's symptoms of severe nausea, vomiting of undigested food, post-meal heartburn, and early satiety, which are indicative of a condition affecting the digestive process. The patient's longstanding history of type 2 diabetes is a significant factor in this diagnosis. Diabetes can lead to autonomic neuropathy, which affects the nerves in the digestive system and can result in gastroparesis. Gastroparesis is characterized by delayed gastric emptying due to decreased gastric myoelectrical activity. This condition can explain the patient's symptoms, as food stays longer in the stomach, leading to nausea, vomiting of undigested food, and early satiety. The other options provided, including accumulation of substances in the bile ducts (Option A), decreased gastric pH (Option C), and increased cholecystokinin activity (Option D), were ruled out based on the patient's symptoms, lab results, and medical history. These options would likely present with different symptoms or abnormal lab results, which are not present in this case. Therefore, the consensus is that the patient's condition can best be explained by decreased gastric myoelectrical activity, likely indicative of gastroparesis due to diabetic autonomic neuropathy.
